# Chronic atrophic gastritis in different ages in South China: a 10-year retrospective analysis

**DOI:** 10.1186/s12876-023-02662-1

**Published:** 2023-02-09

**Authors:** Zefeng Zhang, Xiaoguang Zhang

**Affiliations:** Department of Digestive EndoscopDepartment of Digestive Endoscopy Center, Guangdong Provincial People’s Hospital (Guangdong Academy of Medical Sciences), Southern Medical University, 106 Zhongshan Second Road, Guangzhou, 510080 Guangdong People’s Republic of China

**Keywords:** Prevalence, Age, Chronic atrophic gastritis, Autoimmune atrophic gastritis, Endoscopy

## Abstract

**Objectives:**

To explore the prevalence, characteristics, age distribution and etiology changes of chronic atrophic gastritis (CAG) in South China.

**Methods:**

This study included all patients who underwent endoscopy examinations from 2011 to 2020 in our hospital. Patients were divided into groups 1 (2011–2015) and 2 (2016–2020). The prevalence, characteristics, age distribution and etiology changes of CAG were compared between groups.

**Results:**

Overall CAG prevalence was 20.92% (24,084/115,110) from 2011 to 2020; prevalence significantly differed between groups (18.78%, 8468/45,087, in group 1 and 22.30%, 15,616/70,023, in group 2). Patients with CAG had significantly younger age (under 45) and more corpus atrophy and more autoimmune atrophic gastritis (AAG) in group 2 than in group 1. AAG prevalence in group 2 was 30.11% (4702/15,616) significantly higher than 13.57% (1149/8468) in group 1. 82 patients with AAG later exhibited gastric cancer without obvious clinical features over the decade.

**Conclusions:**

CAG is increasing and seems starting earlier among people during the study period. We need to focus on diagnosis and treatment of corpus related atrophy and AAG, especially for the young. Laboratory examination, endoscopic biopsy and surveillance are important for CAG.

## Introduction

Chronic atrophic gastritis (CAG), which comprises atrophied and reduced gastric mucosal epithelium and glands, is often closely related to Helicobacter pylori (Hp) infection and expected to increase with age. However, CAG usually lacks specific clinical manifestations, and it remains reliant on pathological diagnosis in China. Endoscopic presentation is critical in determining biopsy sites and increasing the rate of CAG diagnosis [1]. In addition, H.pylori-related CAG has been associated with the occurrence of gastric cancer [2, 3]. Because gastric cancer has an annual incidence of 0.1% among patients who exhibit CAG within the preceding 5 years [4], CAG is generally regarded as a risk factor for gastric cancer.

China is a country with high prevalence of Hp infection and gastric cancer [1]; therefore, early prevention and treatment for CAG can provide substantial social and economic benefit. The prevalence of CAG vary widely among regions and countries, ranging from 4.1% at age 25 to 13.3% at age 99 in Europe and West Asia [5]. To our knowledge, there has only been one nationwide multicenter survey, which enrolled 8892 patients (18–65 years of age) among 10 cities (three southern cities) in China in 2011; it showed that the prevalence of CAG was 25.8% [6]. This finding suggested that China has a high prevalence of CAG, especially in northern and central areas. In our study, we explored the prevalence, characteristics, age distribution and etiology changes of CAG in South China over a 10-year period.

## Methods

All patients who had firstly undergone endoscopy examinations from 1 January 2011 to 31 December 2020 in our hospital were enrolled, with the exception of patients who had upper gastrointestinal tumors and/or bleeding. All patients provided written informed consent to be included in the study prior to their examinations. All patients were taken two biopsies in antrum for both pathology and Hp tests. Biopsies were also collected from red and white mucosal sites in the body for pathology and another biopsy in the body was taken for Hp test when the inflammation was obvious. Formalin-fixed specimens were immediately sent for pathological diagnosis.

CAG was pathologically diagnosed on the basis of atrophied and reduced gastric mucosal epithelium and glands, as well as intestinal metaplasia, in accordance with the chronic gastritis guidelines established in China in 2017 [7]. Samples that had been collected before publication of the guidelines were retrospectively evaluated using the definitions in the 2017 guidelines.

Autoimmune atrophic gastritis (AAG) was defined as including gastric body atrophy, no Helicobacter pylori(Hp) present infection, negative Hp antibody in serum, and one of the three situations (positive intrinsic factor antibody findings, positive parietal cell antibody findings, Pepsinogens I ≤ 70 ng/ml and ratio of Pepsinogens I/Pepsinogens II ≤ 3). Hp present infection was defined as either positive rapid urease test results (Huitai Medical Technology Company, Shanghai, China) or positive pathological findings. Patients with gastric cancer after AAG were further analyzed.

All patients were divided into groups 1 (2011–2015) and 2 (2016–2020). Patients who were diagnosed as CAG in group 1 were not included in group 2. The prevalence, characteristics, age distribution and etiology changes of CAG were compared between groups. All data were presented as counts and percentages. Paired Student’s t-tests and chi-square tests were used to investigate differences between groups. *P*-values < 0.05 were considered statistically significant.

## Results

In total, there were 24,084 patients with CAG during the entire study period; the prevalence of CAG was 20.92% (24,084/115,110). However, the prevalence increased slowly during the study period, such that it was significantly higher in group 2 than in group 1 (*P* < 0.05; Table1). Furthermore, there were 12,605 (52.34%) male patients with CAG and 11,749 (48.78%) female patients with CAG during the entire study period (24,084 total patients). In group 1, there were 4397 (51.92%) male patients with CAG and 4071 (48.08%) female patients with CAG (8468 total patients); in group 2, there were 8208 (52.56%) male patients with CAG and 7408 (47.44%) female patients with CAG (15,616 total patients). The proportions of male and female patients did not significantly differ between groups 1 and 2.

**Table 1:** Age distribution of patients with CAG in South China from 2011 to 2020

Group/Years		11–29*	30–44*	45–59	60–74	75–93	All patients
1 (2011–2015)	CAG	311	1778	3670	2268	441	8468
	Total	5048	12,717	16,118	9239	1965	45,087
	%	6.16%	13.98%	22.77%	24.55%	22.44%	18.78%
2 (2016–2020)	CAG	517	2890	6619	4751	839	15,616
	Total	5144	16,170	27,827	17,587	3295	70,023
	%	10.05%	17.87%	23.78%	27.01%	25.46%	22.30%

*P < 0.05

Overall, the prevalence of CAG increased in all age groups during the study period. CAG mostly occurred in middle-aged and older men (45 years of age); these comprised 69.76% of the patients in group 1 and 76.25% of the patients in group 2 (the proportion did not significantly differ between groups). Furthermore, there were more young patients with CAG (i.e., 11–29 years and 30–44 years) in group 2 than in group 1 (*P* < 0.05; Table 1).

**Fig. 1:**
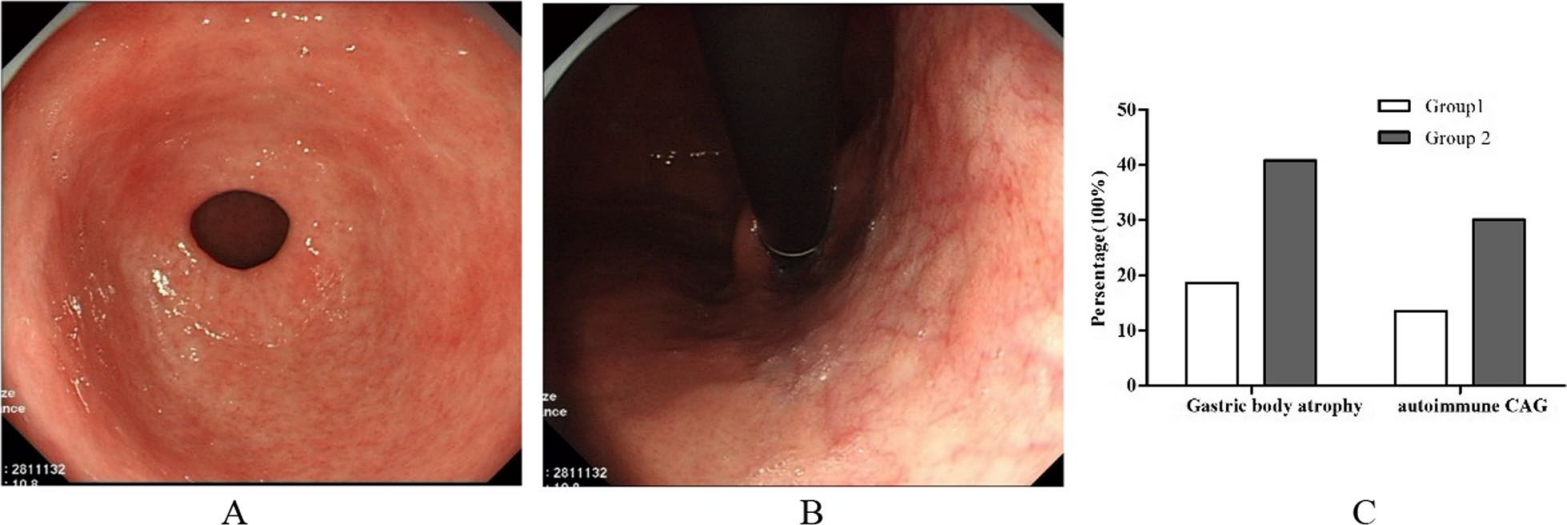
**A**–**C** CAG in the nearly decade in group 1 and group 2; **A** antrum atrophy; **B** corpus atrophy; **C** percentages of corpus atrophy and AAG, *P* < 0.05

In patients with CAG, the gastric mucosa exhibited red and white color in an alternating manner. Furthermore, large portions of mucosa were pale, thin, coarse, and dry, with cracks; the mucosa exhibited lighter color with a clearly visible underlying vascular network, and bile acid reflux was present (Fig. 1A, antrum atrophy; Fig. 1B, corpus atrophy). The pathological degree of atrophy and metaplasia of patients with CAG and AAG were shown in Table 2; there might be less moderate and severe atrophy in CAG in group 2 than group 1 (*P* < 0.05); it appeared that CAG in group 2 consisted largely of mild atrophy. However, it shown no difference in pathological degree of atrophy and metaplasia of patients with AAG between group 1 and group 2. It presented that AAG probably had more percentages of moderate and severe atrophy than CAG in group 2. The prevalence of gastric body atrophy was 40.86% (6380/15,616) in group 2, more than twofold greater than the prevalence in group 1 as 18.64% (1578/8468) (Fig. 1C), suggesting that the prevalence of gastric body atrophy increased during the second half of the study period; this might be related to dietary and environmental changes. In addition, the prevalence of AAG was 30.11% (4702/15,616) in group 2, higher than the prevalence of 13.57% (1149/8468) in group 1 (Fig. 1C) (*P* < 0.05).

**Table 2:** Pathological degree of atrophy and metaplasia of patients with CAG and AAG in South China from 2011 to 2020

Group/Years	Mild atrophy	Moderate atrophy	Severe atrophy	Total atrophy	Metaplasia	Disease
1 (2011–2015)	4493	3367*	608*	8468	6721	CAG
2 (2016–2020)	11,107	3810*	699*	15,616	13,349	CAG
1 (2011–2015)	684	376	89	1149	824	AAG
2 (2016–2020)	2765	1632	305	4702	3194	AAG

*CAG* chronic atrophic gastritis, *AAG* autoimmune atrophic gastritis

*P < 0.05

More importantly, 82 (1.40%, 82/5851) gastric cancer patients after AAG (seen as Table 3), including 19 high-grade intraepithelial neoplasia (HGIEN) or intramucosal carcinoma, 13 gastric neuroendocrine tumor and 50 advanced gastric cancer were totally found in the decade. We further analyze the clinical features, dietary habits and medication before gastric cancer diagnosed. Most patients drank tea or coffee, but had no folic acid or vitamin B12 supplement in their daily life. In most instances, gastric cancers appeared after 2 or more years with AAG.

**Table 3:** Analysis of clinical features in the 82 gastric related cancer patients after AAG

Features		Cases
Gender	Male	45
	Female	37
Age	> 45	56
	< 45	26
Diet	Tea/coffee	63
	No tea/coffee	19
Folic acid	Supplement	25
	No supplement	57
Vitamin B12	Supplement	33
	No supplement	49
Mucosaprotector	Supplement	75
	No supplement	7
Cancer diagnosis time	< 2 year	8
	2 ~ 5 year	25
	5 ~ 10 year	49
Related cancer	HGIEN/ intramucosal carcinoma*	19
	Neuroendocrine tumor*	13
	Advanced gastric cancer^#^	50

*HGIEN or intramucosal carcinoma and gastric neuroendocrine tumor, were performed ESD and semiannual gastroscopy examination after ESD

^#^Advanced gastric cancer, were performed surgery and/or radiochemotherapy

Endoscopic submucosal dissection (ESD) is suitable and useful for HGIEN, intramucosal carcinoma, and gastric neuroendocrine tumor; since no tumor recurrence occurs in the follow-up.

## Discussion

CAG and intestinal metaplasia have been identified as independent risk factors for gastric cancer [8]. In a study of 405,172 patients in Sweden, the prevalence of gastric cancer was 4.5-fold greater among patients with CAG than among individuals without CAG [9]. This finding suggested that CAG comprises a pre-gastric cancer state, closely associated with the onset of gastric cancer. Gastric biopsies, expressed as Sydney system grade and OLGA/OLGIM classifications, represent the gold standard for diagnosis and cancer risk stage [10]. H. pylori infection, intestinal metaplasia, older age, drinking, pernicious anemia, and moderate to severe CAG are considered high risk factors for CAG progression to gastric cancer [11, 12]. CAG is relatively common among older adults in different parts of the world, but large variations exist in different reports up to now [13].

China has a high prevalence of CAG, as indicated in a previous study [1]. Our current study showed that the prevalence of CAG is increasing in South China. In addition, CAG increasingly occurred among younger individuals (especially in patients aged < 45 years), indicating that it is a multifactorial disease and age may be not the leading cause. There is a need to identify additional risk factors for the pathogenesis of CAG, including aspects of Helicobacter pylori infection. Since H.pylori-related CAG has a long clinical course and H. pylori eradication may be beneficial by modifying the natural history of atrophy, we continue to recommend quadruple therapy within 14 days after diagnosis to interfere with CAG development as soon as possible; and timely assessment after treatment is needed to confirm clinical effects. Helicobacter pylori-related CAG and AAG are regarded as two different diseases, but they display overlapping features [13].

In our study, the rate of AAG showed increasingly among patients in group 2 than group 1. This suggests that the overall Hp infection rate might be declining, presumably because of the recent focus on Helicobacter pylori eradication in China. Furthermore, the youngest patient with CAG was only 11 years of age; a small number of patients with CAG were under 30 years of age. Notably, more corpus atrophy and AAG were observed among patients in group 2 than among patients in group 1.

AAG, characterized by the destruction of gastric parietal cells and leading to the loss of intrinsic factor and reduced acid output [14], is increasing and attracting more attention nowadays. AAG is often delayed diagnosed due to its various insignificant and nonspecific clinical features, and uncommon vitamin B12 deficiency-related manifestations in female are overlooked [15]. AAG seems to be common in normal weight, dyspeptic women with iron-deficiency anemia and autoimmune thyroid disease, and in overweight male smokers with pernicious anemia [16]. Anaemia is a important manifestation in AAG patients, mostly due to vitamin B12 deficiency [17], which results in a megaloblastic anemia and iron malabsorption, leading to iron deficiency anemia. In the last years the deficiency of several other vitamins and micronutrients, such as vitamin C [18], folic acid [18], 25-OH vitamin D [17] and impairment of vitamin D absorption [19], has been increasingly described in patients with AAG. However, the underlying shared pathogenic mechanisms still need to be further studied. Scant haematologic alterations and micronutrient deficiencies may precede overt anaemia [20]. Early histopathological alterations allowing a more precise and prompt recognition for diagnosis for AAG have recently been reported. It was recently described that roughly 20% of patients were seronegative at the time of AAG histological diagnosis, especially in elderly individuals [21]. Sometimes, AAG patients are misdiagnosed as refractory to H pylori eradication therapy, probably because achlorhydria might allow urease-positive bacteria other than H pylori to colonize the stomach, causing positive 13C-urea breath test results [22]. In addition, a gastroscopy examination is suggested for those with a concurrent autoimmune disorder within 2 years [23]. AAG might finally develop into neuroendocrine tumors and gastric adenocarcinoma. Management includes early detection through a proactive case-finding strategy, micronutrient supplementation and endoscopic surveillance are helpful and essential for AAG [14]. CAG usually occurs without overt clinical signs in daily life. Most patients (especially young individuals) do not readily undergo medical examinations such as endoscopy. So it is often delayed diagnosed and may lead to a lower recorded prevalence of CAG, compared with the actual incidence [24]. We advise gastroenterologists to carefully observe suspected lesions in the corpus and antrum. Electronic chromoendoscopy is suggested for the "targeted biopsies" of intestinal metaplasia [10]. Pepsinogens (Pepsinogens I and II) and gastrin-17, may help to identify atrophic locations; intrinsic factor antibody and parietal cell antibody tests and Helicobacter pylori testsmay aid in determining the cause among CAG patients. Laboratory and endoscopic examination are both important for screening and diagnosis of CAG.

Patients with advanced stages of CAG (Stage III/IV OLGA or OLGIM) should undergo endoscopic surveillance every three years, those with AAG every three-five years [13]. In our study, totally 82 patients after AAG later exhibited gastric cancer without obvious clinical features; the mean progression interval was approximately 5 years. We agree that the first endoscopic surveillance 3 years after diagnosis seems safe for AAG [25]. Since AAG is a steadily progressive disease, we should focus on the patients with more severe gastric lesions [26]. Follow-up endoscopy and biopsies provide the possibility of early diagnosis and operation chance for patients. It is worth mentioning that postoperative management should be performed the first follow-up gastroscopy within 12 months.

There were some limitations in this study. Because it was a retrospective analysis with a large number of patients, there might have been subjective differences among endoscopists and pathologists. Furthermore, we could not calculate the precise number of patients with CAG who later developed gastric cancer, nor could we determine the prognoses and clinical outcomes of these patients. More interestingly, the latest opinion that corpus-restricted atrophy does not increase the gastric cancer risk and the excess of gastric cancer risk reported in patients with AAG could plausibly result from unrecognised previous/current H. pylori comorbidity [27] has come to our notice. These points will be the main focus of our research in future studies.

## Data Availability

The data that support the findings of this study are available from Department of Digestive Endoscopy Center, Guangdong Provincial People’s Hospital, but restrictions apply to the availability of these data, which were used under license for the current study, and so are not publicly available. Data are however available from the corresponding author upon reasonable request and with permission of Department of Digestive Endoscopy Center, Guangdong Provincial People’s Hospital.
